# How instructions modify perception: An fMRI study investigating brain areas involved in attributing human agency

**DOI:** 10.1016/j.neuroimage.2010.04.025

**Published:** 2010-08-01

**Authors:** James Stanley, Emma Gowen, R. Christopher Miall

**Affiliations:** a Department of Public Health, University of Otago, Wellington, New Zealand; b Faculty of Life Sciences, Moffat Building, The University of Manchester, PO Box 88, Sackville Street, Manchester, M60 1QD, UK; c Behavioural Brain Sciences, School of Psychology, University of Birmingham, UK

**Keywords:** Biological motion, Paracingulate cortex, Mirror neurons, Superior temporal sulcus, Agency

## Abstract

Behavioural studies suggest that the processing of movement stimuli is influenced by beliefs about the agency behind these actions. The current study examined how activity in social and action related brain areas differs when participants were instructed that identical movement stimuli were either human or computer generated. Participants viewed a series of point-light animation figures derived from motion-capture recordings of a moving actor, while functional magnetic resonance imaging (fMRI) was used to monitor patterns of neural activity. The stimuli were scrambled to produce a range of stimulus realism categories; furthermore, before each trial participants were told that they were about to view either a recording of human movement or a computer-simulated pattern of movement. Behavioural results suggested that agency instructions influenced participants' perceptions of the stimuli. The fMRI analysis indicated different functions within the paracingulate cortex: ventral paracingulate cortex was more active for human compared to computer agency instructed trials across all stimulus types, whereas dorsal paracingulate cortex was activated more highly in conflicting conditions (human instruction, low realism or vice versa). These findings support the hypothesis that ventral paracingulate encodes stimuli deemed to be of human origin, whereas dorsal paracingulate cortex is involved more in the ascertainment of human or intentional agency during the observation of ambiguous stimuli. Our results highlight the importance of prior instructions or beliefs on movement processing and the role of the paracingulate cortex in integrating prior knowledge with bottom-up stimuli.

## Introduction

Perception and performance of action are closely entwined neural processes. For example, speed of action initiation can be facilitated by the concurrent viewing of a congruent action, or can be inhibited if an incongruent action is observed at the same time as performance (Brass et al., 2001; Brass et al., 2000; Craighero et al., 2002; Press, et al., 2005; Vogt et al., 2003). More complex aspects of action performance are also influenced by simultaneous observation of a matching or mismatching action. Kilner et al. (2003) showed that performance of horizontal or vertical arm movement is influenced by the simultaneous observation of someone moving their arm in the perpendicular plane, hereafter termed ‘movement interference’. An individual observing someone else performing a vertical arm movement will perform horizontal arm movements that have a stronger vertical component than if the observed action was also horizontal — i.e., congruent with the performed action (Kilner et al., 2003); see also Bouquet et al. (2007), Gowen et al. (2008) and Stanley et al. (2007)). Kilner et al. (2003; see also Blakemore and Frith (2005)) suggested that their interference effect was because observed and performed actions lead to coactivation of premotor areas resulting in a motor output that blended the two action profiles. This hypothesis was based on the existence of neural networks involved in both performance and observation processes, now known as “mirror neurons”, in area F5 of the monkey brain. This region (considered analogous to the ventral premotor cortex or BA 44 in the human brain (Binkofski and Buccino, 2004)) is activated both during the performance of an action as well as during passive viewing of the same action (Rizzolatti et al., 1996; see Rizzolatti and Craighero (2004), for a comprehensive review). Subsequent experiments in humans using magnetic resonance imaging (fMRI) have provided support to the hypothesis that passive perception of action involves the ventral premotor cortex and inferior parietal lobe (IPL) that were predominantly thought to be responsible for action/motor performance (Buccino et al., 2004; Grezes and Decety, 2001; Iacoboni et al., 1999; Iacoboni, 2005).

Previous studies highlight behavioural and neural differences when observing human compared with non-biological stimuli. For example, facilitation of movement initiation is less prominent when observing moving robots or mechanical objects (Brass et al., 2001; Craighero et al., 2002; Jonas et al., 2007; Press et al., 2005; Tsai and Brass, 2007). Mirror neuron activity also appears to be stronger when stimuli possess a human rather than artificial form (Chaminade et al., 2007; Rizzolatti and Craighero, 2004), and activation of mirror neuron areas in parietal and premotor cortex is reduced during observation of robotic or virtual-reality limb movements, compared to viewing a person (Engel et al., 2008a; Perani et al., 2001; Tai et al., 2004; although see (Oberman et al., 2008). Activity within the superior temporal sulcus (STS), which responds specifically to observation of human action (Grezes and Decety, 2001; Kourtzi and Kanwisher, 2000; Ruby and Decety, 2001; Senior et al., 2000) is likewise enhanced for viewing real as opposed to cartoon people in videos with identical kinematics (Mar et al., 2007).

Most of these studies have used unambiguous stimuli – clearly identified as either human or non-human movement – to study behavioural and neural effects of observing movement. However, it is unclear to what degree these effects are influenced by the underlying human movement kinematics or the declarative knowledge that a human agent is performing the task. The current experiment aimed to investigate the neural mechanisms of how belief or knowledge about the agency of an observed action can influence the perception of such a stimulus. This question can be reductively phrased as that of bottom-up versus top-down processes — in other words, do the underlying kinematic properties of people acting lead us to perceive a person moving, or does the knowledge that we are watching a person prepare our brain to use systems like the mirror neuron system to understand what we see? One candidate brain area that may be important for top-down control is the paracingulate cortex. This area is preferentially active during interactions with or observation of human compared to non-biological stimuli. For example, greater activation occurs when predicting human as compared to computer actions (Ramnani and Miall, 2003) or playing strategic games against human compared to computer agents (Gallagher et al., 2002; McCabe et al., 2001). These studies suggest that the paracingulate cortex plays a role in preferentially processing human actions or responses, particularly during tasks that explicitly cue participants to the human nature of the behaviour.

Behavioural studies of movement interference have explored the relative contribution of top-down and bottom-up processes using non-biological or ambiguous stimuli. For example, Kilner et al. (2003) showed that viewing a robotic arm produces no interference effect for a performed action, in contrast to the effect seen when viewing an actual person perform an action. This differential effect may be due to differences in kinematic profile between these two conditions (principally smoothness of acceleration) or due to the presence/absence of a human performer adding agency to the observed action. Using a more humanoid robot instead of a robotic arm, Oztop et al. (2004) did observe an interference effect implying that the robot and human movement were close enough for participants to adopt similar processing strategies for both. Kilner et al. (2007) have since shown that both agency and motion profile impact upon interference effects.

Our own work suggests that top-down processes regarding action agency play a major part in the interference effect for an ambiguous stimulus — a moving dot projected onto a screen. In a paradigm adapted from Kilner et al.'s (2003) study, participants who were told that the dot was a recording of human movement showed a significant interference effect on performed movements, while another group of participants who were told that the identical dot movement was computer generated showed no such effect (Gowen et al., 2008; Stanley et al., 2007). These results suggest that the interference effect reported in the above studies is strongly dependent on beliefs or instructions about the agency of the observed action, especially in situations where the visual stimulus is ambiguous.

The present experiment was designed to answer how instructions or beliefs regarding agency influence perception of action, and also to identify the corresponding brain areas involved. We employed a range of point-light movement animations together with categorical instructions to influence beliefs regarding the movement type. Point-light animations typically consist of a sequence of moving dots, representing several cardinal joints on an actor's body during the performance of an action (e.g., walking). Viewing these kinematic recordings is enough to not only evoke subjective perception of human movement (Johansson, 1973; see Blake and Shiffrar (2007), for a review) but also to activate neural areas such as the STS and temporal and occipital cortex that are activated during the observation of “real” movement (Bonda et al., 1996; Grossman et al., 2000; Puce and Perrett, 2003). Manipulating the spatio-temporal relationship between the dots (in essence, scrambling the presented stimulus) reduces both subjective perception of human movement as well as neural activity in the STS (Bonda et al., 1996; Grossman et al., 2000). In the current experiment, the point-light animations ranged from those that strongly resembled a person moving to scrambled forms that had little resemblance to a human action and participants were asked to judge whether they perceived the stimuli as containing human movement. To influence beliefs about agency, participants were informed that some stimuli were based on actual recordings of human movement, whereas other stimuli were computer generated patterns of dot movement trials.

A pilot study (see Supplementary material I), confirmed that both the level of visual scrambling and the human/computer category applied to a particular animation would influence subjective responding, with higher likelihood of responding “human-like” for trials labelled human, and for trials that were towards the less-scrambled end of the spectrum. The aim of the fMRI experiment was to investigate the neural substrates of this phenomenon: we hypothesised that if cortical areas such as the paracingulate cortex are implicated in determination of agency they would be differentially activated (with greater BOLD signal) during trials where the participant was told the stimulus was a recording of a person moving. Areas dedicated to the perception of human movement such as ventral premotor cortex, IPL, and STS might also show greater activation in the “human instruction” condition. Such findings would indicate that prior knowledge regarding the agency of a stimulus can prime brain areas to process stimuli in a manner consistent with the agency of movement.

## Materials and methods

### Participants

The participants were 14 (7 female) healthy undergraduate and postgraduate students at the University of Birmingham, U.K. Mean age was 20.5 years (median 19, range 18 to 29). All participants were right-handed, and had either normal visual acuity or used appropriate corrective lenses in the scanner. On completion of the study each participant received either £25 or credit towards a Department of Psychology research participation scheme. Each participant gave written informed consent and the study was approved by a local research ethics committee.

### Stimulus materials

Animations were created specifically for use in this experiment, and six movements were selected: walking, kicking a ball, throwing a ball, throwing a ball underarm, punching, and lifting a small box (a further two movements, including a simulated tennis shot and stepping onto a small platform were used as stimuli in the training section of the experiment but never presented in the main experiment).

The animations were created by recording the actions using a Vicon passive-sensor system (Oxford Metrics Vicon 250) with a six camera setup. Fifteen sensors were attached to the model's body, and position information was recorded at 120 Hz. From these recordings, two-second segments of movement were selected that included clear views of all sensors.

Position information for these movements was processed using Matlab v. 7 (R14, Mathworks Natick, Massachusetts). Movements were converted into 2d space — this included de-trending the position information in the anterior–posterior axis of movement, so that actions that included gross whole body translation (running, walking, and kicking a ball) appeared as though the actor was moving “on the spot”. Position information was then filtered with a 10 Hz low-pass Butterworth filter, and resampled to 60 Hz (to match the frame rate on the projector used), and all sensor coordinates were translated to position relative to the sensor at the middle of the actor's waist. These data served as the “source action files”.

In order to create the stimuli used in the experiment, these source movement files were then altered to create a “random starting position file”. Firstly, a randomization algorithm (conducted in Matlab) randomly allocated a new starting position for each sensor. Possible starting positions were limited by a rectangular prism in 3d space based on the axes of the actor's body when at rest. These positions were generated once for each movement, and are hereafter called the “random starting position movement files”. The translation of each sensor during the action was therefore identical to the source action, but these translations were now relative to the new starting position.

Stimuli were then generated from these two sets of files: position of each dot sensor in the visual display was a weighted average of the Cartesian coordinates of that sensor in the source movement file and the random starting position file. These stimuli are described hereafter by the weighting proportion of the original movement in the final stimulus: for example, a 1.0 stimulus would be the actual source movement stimulus, and a 0.0 stimulus would consist of the random starting position stimulus. A 0.5 stimulus had sensor starting positions that were presented at the mean of the starting position in the source and random files. Six different weighted proportions of the original recording were used^3^: 0.35, 0.45, 0.55, 0.65, 0.75, and 0.85, chosen to represent a range of animations that varied from generally uninterpretable patterns of dot movement (at the lower weighting) to appearing like realistic human movement (at the higher weighting proportions).

Over the course of all the blocks of the main task, each stimulus was presented from one of three viewing angles, calculated prior to converting the stimuli to a 2d model (− 45°, 0°, 45°). Each movement was therefore viewed a total of 36 times: three angles in six different possible mixes of original/random starting position, and with two categories (human or computer).

### Behavioural task

The behavioural task completed by the participants while in the scanner consisted of rating whether the point-light animations looked like human movement or like random moving dots. The task instructions (see Supplementary material II) told the participant that he or she would be viewing two types of animations, both featuring a set of moving dots: the first type of animation consisting of recordings of human movement, and the second type consisting of a computer generated random movement. Stimuli were categorised as either ‘human movement’ or ‘computer generated’: in fact, stimuli were identical in both of these conditions (see Trial organization for details of trial order randomization). The participant was required to rate agreement between the agency category and his or her perception of the animation. Task descriptions emphasized the subjective nature of the participant's perception of the stimuli. On each trial, there were four possible responses as to whether the participant's perception agreed with the category — strongly agree, slightly agree, slightly disagree, and strongly disagree. For the purposes of analysing the behavioural data, these responses were simplified into binary agree/disagree categories.

### Trial organization

For the event-related trial order for the main task, several parameters were kept in place while determining run structure and trial order. Firstly, no two stimuli of the same type were ever presented in the same run. Secondly, within a run no two trials of the same movement pattern (e.g. running, and kicking a ball) were presented in a row. No more than 2 trials of the same category (human or computer) could be presented in a row, and no more than 2 trials of the same realism level (e.g. 0.35 proportion of original movement in displayed animation) could be presented in a row.

A single trial consisted of the following components:

1) An inter-trial interval (ITI) of between 3 and 5 s, during which only a central fixation point was presented onscreen.
2) Presentation of a single letter indicating trial category (H for a human-labelled trial, C for a computer labelled trial) for a semi-random duration of between 3 and 5 s. The durations of the inter-trial interval and pre-trial category were determined so that the onset of the actual animation was jittered relative to the start of fMRI volume collection.
3) The trial category cue remained onscreen during presentation of the main animation. The participant was asked to fixate on this cue during the trial, in order to prevent large scale eye movements during the presentation of the animation. Animation duration was always 2 s.
4) Following presentation of the main animation, the trial category remained onscreen for between 0.75 and 1.25 s. Participants were instructed to fixate during this period and to withhold any response.
5) Following this delay, a rating screen reminder was displayed for a duration of 2 s. This screen consisted of a reminder of the function of the four button response device, and consisted of the following symbols spaced from left to right “−−”, “−”, “+”, and “++”. Participants were instructed to press the button corresponding to their perception of the stimulus (relative to the given category) once this screen appeared.
6) Start of next trial sequence begins with ITI (step 1).

### Experimental procedure

Each participant completed the experiment over two days: on one day, the participant completed the main task, while on the second day he or she completed a localizer task as well as a structural scan. Session order was counterbalanced between subjects (half completed the main experiment first, then localizer/structural; the other half completed the localizer/structural first, and then the main experiment).

Prior to performing the main experiment, each participant completed a set of practice trials to familiarize him or her with the trial structure and required responses. During this period the experimenter corrected the participant's responses if necessary (by asking whether the participant's button press corresponded to his/her intended response regarding agreement/disagreement), and answered other questions where possible. To prevent excess familiarity with the animations, the two point-light animations used for these practice trials were not used in the main experiment.

The localizer task consisted of viewing a series of short point-light animations in quick succession. A total of ten animations were presented, each lasting for 0.55 s: point-light animations of walking, running, boxing, jumping, and marching, and scrambled (with respect to each dot's starting position) versions of these five stimuli. The participant had to respond with a single button press if the animation they had just viewed was identical to the previous animation. No response was required in the case of mismatches. Animations were organized into 20 second blocks: the human movement blocks and scrambled blocks contained six animations in a row (with a 0.55 second ITI between each animation, where responses were required for matching animations), while the baseline blocks consisted of 20 s of fixation. Each participant completed two runs of trials, each lasting for 315 s. This task and set of stimuli has been used previously for the purposes of localizing neural areas involved in the perception of human action (Grossman and Blake, 2002).

Each run of the localizer task consisted of 7 sets of human movement (six animations in each set), 7 sets of scrambled movement (six animations in each set), and 5 sets of fixation (18 second duration, that is 3 TR, to serve as baseline).

### Scanner details

The fMRI sessions were conducted using a Philips 3 T MRI scanner with SENSE headcoil. Participants viewed the stimuli on a back-projected screen located at the rear of the scanner core. For the functional scanning sessions (including the localizer), volumes were taken with a voxel size of 2.5 × 3 × 3 mm. The main task session was divided into six runs, each consisting of 150 volumes (with a TR of 3 s), giving a duration of 7.5 min per run. Prior to the start of the run the fMRI scanner took 15 s (5 volumes) to achieve T2 equilibrium/saturation. Each run contained 3 stimuli from each stimulus realism/agency category, making for a total of 36 stimuli presented in a single run. The experiment was set up on an event-related schedule (as opposed to a block design), so that trials from each condition were interspersed with one another throughout each run.

The localizer task sessions were performed using the same voxel dimensions and TR as the main task: two runs of trials were presented, each lasting for 105 volumes (a duration of 5 min 15 s per run). A high resolution structural scan was taken in the same session, following the localizer task, with a resolution of 1 × 1 × 1 mm.

### fMRI signal processing and data analysis

Data were analysed using the FMRIB Software Library (FSL) package (FSL v. 3.3, FMRIB, Oxford University; for details see Woolrich et al. (2009) http://www.fmrib.ox.ac.uk/fsl/). Prior to analysis, a slice-timing correction was applied to the functional data, which were then motion corrected using the MCFLIRT algorithm in FSL (Jenkinson and Smith, 2001). Data analysis was broken into three levels. At level 1, analysis was completed for each recording run. Individual trial timings from the Presentation logfiles were fit to the functional data in order to calculate trial onset relative to scanner volume onset. The model at level one consisted of onset and duration of the animation stimuli, grouped by agency category (2 levels) and stimulus realism (6 levels) — no distinction was made between the different actions or the different perspective angles. All other phases of the trials were unmodelled (baseline or rest). Motion correction parameters (derived from MCFLIRT) were also included in the model as covariates of no interest. Functional data were registered to a Montreal Neurologic Institute (MNI) standard brain image. Contrasts were calculated looking at the main effect of stimulus category (human or computer category trial), and main linear effect of stimulus realism (here using the more detailed categories of 0.35, 0.45, 0.55, 0.65, 0.75 and 0.85 proportions of the original movement locations). Positive and negative interaction terms between these two factors were also calculated: these interactions can be considered as testing for areas more active when stimulus and label were congruent (higher realism with human label, or lower realism with computer label) in the case of the positive interaction, and for areas that are more active when stimulus and label were incongruent (higher realism with computer label, or lower realism with human label) in the case of the negative interaction.

Level 2 of the functional analysis involved a fixed-effects combination of runs for each participant, providing a participant-by-participant average of the Level 1 contrasts. The output of this level was then used as the basis for the third level of analysis, where results were combined using FLAME level 3 analysis within FEAT (Beckmann et al., 2003), which in effect combines responses across participants using random-effects modelling. The contrasts from this final model were thresholded at *Z* > 2.3, with an adjusted cluster threshold of *p* < .05.

In order to describe the neural activations discovered by the principal data analysis, clusters of significant activity found from the group analysis were matched anatomically using comparisons between the 3dmrx (MRIcro) voxel categorised Brodmann atlas, an atlas for general neuroanatomical reference (Duvernoy et al., 1999) and one for localisation within the cerebellum (Schmahmann and Toga, 2000). In order to identify regions of interest such as the inferior parietal area, paracingulate cortex and ventral premotor cortex considered *a priori* to be likely to show differences in activity between the agency category and stimulus realism categories, clusters of significant activity were compared to reported activation coordinates for these regions (Engel et al., 2008a,b; Grezes et al., 2003; Perani et al., 2001; Ramnani and Miall, 2003; Tavares et al., 2008). From the group average signal, local maxima within these areas were compared across the 6 different stimulus realism categories for computer and human instruction using the Featquery tool (FMRIB, Oxford; see the FSL website for details: http://www.fmrib.ox.ac.uk/fsl/feat5/featquery.html).

A mask for each region of interest was created for each participant. The mask area was based on the 3dmrx Brodmann map transformed to the subject-specific structural image, with all voxels within the cluster selected that fell within the anatomically defined region. Using Featquery (an FSL tool) this anatomically defined cluster mask was then applied to extract the mean activation for the subject-specific functional data, representing the % change in BOLD signal during each task period, relative to the mean signal intensity in that area during the unmodelled baseline period (rest). As this unmodelled period includes visual and response components (jittered relative to the onset of the point-light stimuli), the exact meaning of “zero” percentage change in these descriptive analyses should be considered arbitrary — the important elements are the relative differences between stimuli categories.

### Analysis of localizer task

Analysis of the localizer dataset was performed using FSL using a block design. Slice-timing correction and motion correction were performed on data from individual runs prior to analysis. FSL analysis was performed over three levels. At the first level, the human and scrambled movement blocks were modelled, with the fixation periods serving as baseline periods. Contrasts were then calculated comparing the human and scrambled movement blocks. The second level combined the two runs for each participant, using a fixed-effects analysis. Thresholds for activation were set at *Z* > 2.3, cluster *p* < .05. This threshold map was used to create region of interest (ROI) masks for the region of the superior temporal sulcus (STS): the group-wise activations in this task were also calculated by combining each participant's level 2 Feat analyses, using full Flame modelling in FSL to calculate overall activations across all participants.

The localizer task provided the basis for making participant-specific ROI masks for the STS. The analysis of the localizer task produced a single contrast of interest for each individual for areas showing greater activity during human movement blocks than during scrambled movement blocks. Using these contrasts, two observers (authors EG and JS) described ROIs for each participant for significant clusters of activity in the right STS. These ROIs were manually checked for agreement, and then added to form one STS ROI for each participant. Where agreement was poor between these two observers (a total of 3 participants), a third observer (RCM) created a ROI, and of the three ROIs created, the two closest in agreement were combined. These ROI were used to look at activity in the right hemisphere STS for the main task.

### Statistical analysis of behavioural data

Behavioural data were transformed prior to analysis. To simplify analysis (and increase power), response data were collapsed into three categories of stimulus realism: low realism (0.35 and 0.45 stimuli); medium realism (0.55 and 0.65 stimuli); and high realism (0.75 and 0.85 stimuli). Responses were then binarised into “agree”/”disagree” categories from the four original button codes (strongly agree, slightly agree, slightly disagree, and strongly disagree). Agreement score code was inverted for the “Computer” category trials so that all proportions reported are now percentage of trials where participant rated the stimulus as looking more like a person moving than a random collection of moving dots.

These proportional data were then logit-transformed – $\ln\left(\frac{p}{\left(1-p\right)}\right)$ – to make them more suitable for analysis using general linear model techniques (for a general discussion of the logit transformation in the context of signal detection, see DeCarlo (1998)). Data were fit into a two-way repeated-measures ANOVA, with Category as one factor (human or computer category stimulus), and stimulus realism as the second factor (low, medium, and high human realism stimuli). Significant main effects and interactions were followed up with paired *t*-tests as appropriate.^4^ Averages across participants were converted back to the original proportion scale for graphical presentation: confidence intervals were calculated on the logit-transformed data, and again were converted back to the proportion scale for graphical presentation.

## Results

### Behavioural results

The repeated-measures ANOVA of the logit-transformed response data indicated a non significant trend for Category, *F*(1,13) = 3.51, *p* = .084. Stimuli were more often reported as “human” if categorised accordingly (Table 1).

The main effect for “Level” was significant, *F*(2,26) = 324.29, *p* < .001. As the level of animation order increased, participants were more likely to rate the animation as “human”, again shown in Table 1.

**Fig. 1:**
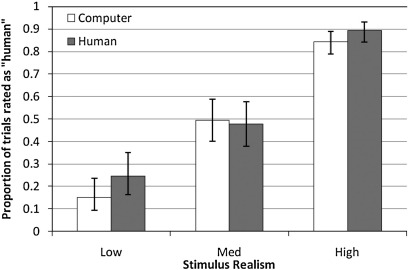
Proportions of trials rated as “looks more like a person moving than random”. Error bars show 95% confidence intervals (calculated on logit scale).

There was also a significant interaction between Category and Level, *F*(2,26) = 6.54, *p* = .005. These data are presented in Fig. 1 (with mean and 95% confidence intervals presented as proportions). Follow up paired *t*-tests were performed on these data, indicating that the difference between human and computer categorised stimuli was most significant for the “low realism” group, *t*(13) = 2.92, *p* = .012. Animations in the “human” category were more likely to be rated as “human” in this realism condition. At the medium realism level, response probability did not differ between the two categories, *t*(13) = 0.42, *p* = .678. The difference at the “highly realistic” stimuli approached significance, *t*(13) = 1.87, *p* = .084, again with a trend for more animations to be rated as “yes, looks like a person” when the category was human than when the category was computer.

#### fMRI data

##### Localiser task

The localiser task confirmed that, across the entire study group, right hemisphere STS activation was enhanced during the normal animations compared to the scrambled animations (see Supplementary material III). These results allowed us to identify any areas of STS activity in the following contrasts.

##### Main linear effect of stimulus realism

As expected, brain areas previously associated with biological motion increased in activity as stimulus realism increased across the 6 levels including right and left primary and extrastriate visual cortices (BA 17, BA 19 and right BA 18), right and left fusiform gyri (BA 37) and left cerebellar lobule VI (Table 2). Right STS activity was also present at a lower significance value of *Z* = 2.9, (*x* = 46; *y* = − 30; *z* = 2). The only area that displayed increased activity with decreasing stimulus realism was right primary visual cortex (Table 3).

##### Effect of instruction

**Fig. 2:**
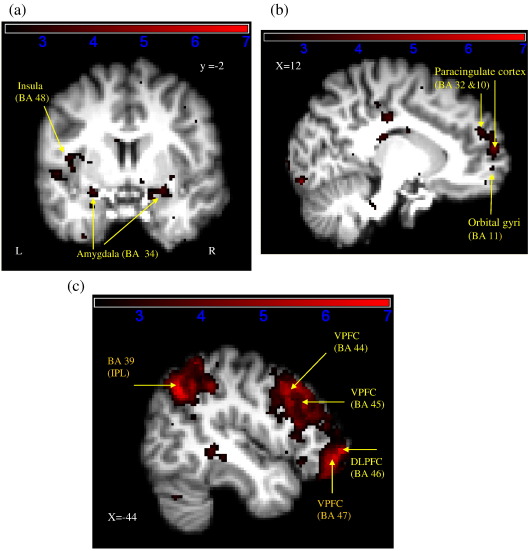
Activation map detailing areas of greater activity during human compared to computer instruction (a–b) and computer compared to human instruction (c) based on group data. Coordinates in MNI space are indicated on each figure. Colour bars indicate *Z* score significance level, from 2.3 (black) to 7 (red). VPFC = ventral prefrontal cortex, IPL = inferior parietal lobe, DLPFC = dorsolateral prefrontal cortex.

The comparison between the human and computer instructions revealed different activation patterns for each condition. The human instruction category resulted in greater activation in right and left visual cortices (BA 17 and 18), left medial temporal areas such as the parahippocampus (BA 36) and hippocampus (BA 20), the left amygdala and left insula cortex and Rolandic operculum (BA 48) (Fig 2a). In addition, the right dorsal and ventral paracingulate cortex (BA 10 and 32) and orbital gyrus (BA 11) were also more active than in the computer instruction trials (Fig 2b). However, contrary to our predictions, increased STS and ventral premotor cortex activity was not apparent in this contrast. These results are summarised in Table 4.

The computer instruction was associated with highly significant activation in right and left ventral prefrontal cortices (BA 44, 45 and 47), left DLPFC (BA 46), and left and right inferior and superior parietal lobes (BA 7 and 39) (Fig 2c). In addition, right cerebellum (crus 1 and lobule VIIIA) and areas of the left inferior temporal gyrus (BA 20 and 37), middle temporal gyrus (BA 21) and lingual gyrus (BA 37) were more active for this category. A list of activation clusters for the computer minus human trials contrast is presented in Table 5.

##### Stimulus–instruction interactions

**Fig. 3:**
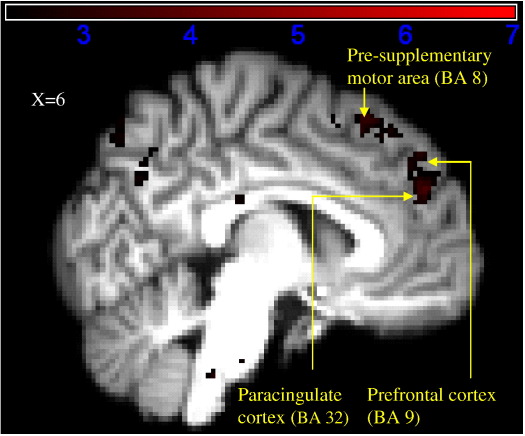
Activation map detailing areas showing greater activation when human instruction and stimulus realism were incongruent. Coordinates in MNI space are indicated on each figure. Colour bars indicate *Z* score significance level, from the lowest score of 2.3 (black) to the highest score of 7 (red).

Brain areas more active when the stimulus and category were inconsistent (e.g. a low realism stimulus with a human trial category, or a high realism stimulus with a computer trial category) are displayed in Fig. 3 and Table 6, with active areas from this interaction being localized to the right hemisphere. Activity was greater in the prefrontal cortex (BA 9), dorsal paracingulate cortex (BA 10), pre-supplementary area, inferior parietal (BA 39 and 40) and superior parietal (BA 7) lobes.

Table 7 documents the opposite interaction where activation was greater when stimuli and instructions were consistent. Left anterior parietal areas (BA 2 and 3), primary motor cortex and supplementary motor area (BA 6) along with right and left visual cortices (BA 18 and 19) were more active. Additional areas included the right superior temporal lobe (BA 22), left middle temporal gyrus (BA 37), right insula cortex (BA 48) and left superior parietal lobe (BA 7). Right STS activity was also present when instruction and stimuli were congruent, at a significance level of *Z* = 2.73 (*x* = 52, *y* = − 42, *z* = 14).

##### Region of interest analysis

We examined how regions of interests identified from the above contrasts varied in activity over the different levels of stimulus realism and instruction using Featquery (see Materials and methods). To simplify data presentation, stimulus realism was collapsed into low, medium and high as with the behavioural data. Percentage change indicates change relative to the mean level of unmodelled baseline (rest) activity.

**Fig. 4:**
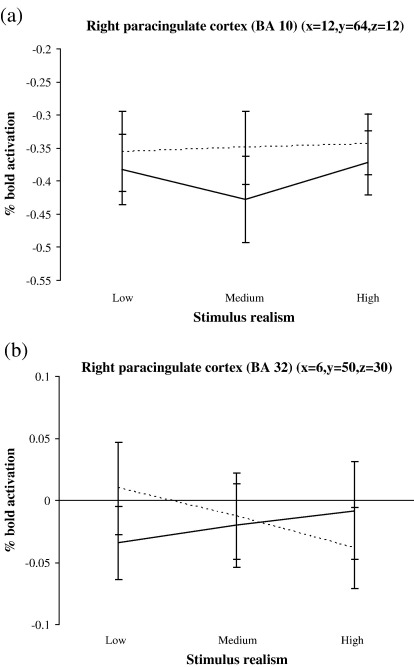
Bold activation % change (relative to baseline mean intensity) for the right paracingulate cortex (BA 10) (a) and BA 32 (b) over the different stimulus realism conditions. Dotted line indicates human instruction, solid line indicates computer instruction. Stimulus realism has been collapsed into low, medium and high. Negative values indicate the mean level of activation in that region was lower than during the baseline period. Standard error bars are shown.

In regards to the two regions of the paracingulate cortex that were identified, BA 10 activity was greater for the human instruction across all conditions (Fig. 4a) whereas activity in the more dorsal and posterior paracingulate location (BA 32) differed according to stimulus reality and instruction: activity increased and decreased for the computer and human instructions respectively as stimulus realism increased (Fig. 4b). Therefore, the paracingulate cortex appears to respond both to the instructed agency (BA 10 and 32) and to whether there is conflict between instruction and stimuli characteristics (BA 32).

**Fig. 5:**
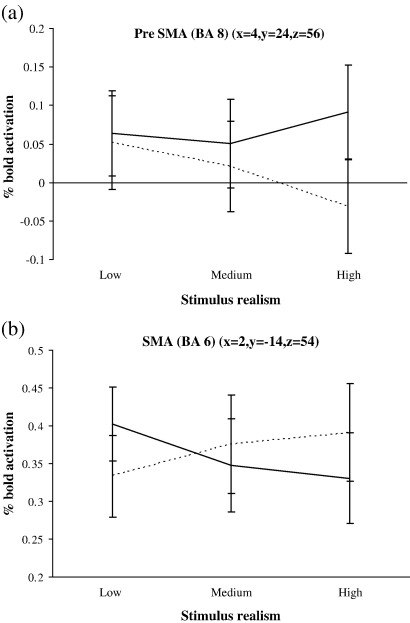
Bold activation % change (relative to baseline mean intensity) for the Pre-SMA (a) and SMA (b) over the different stimulus realism conditions. Dotted line indicates human instruction, solid line indicated computer instruction. Stimulus realism has been collapsed into low, medium and high. Zero indicates activation was equal to the mean level of baseline activity in that region. Standard error bars are shown.

**Fig. 6:**
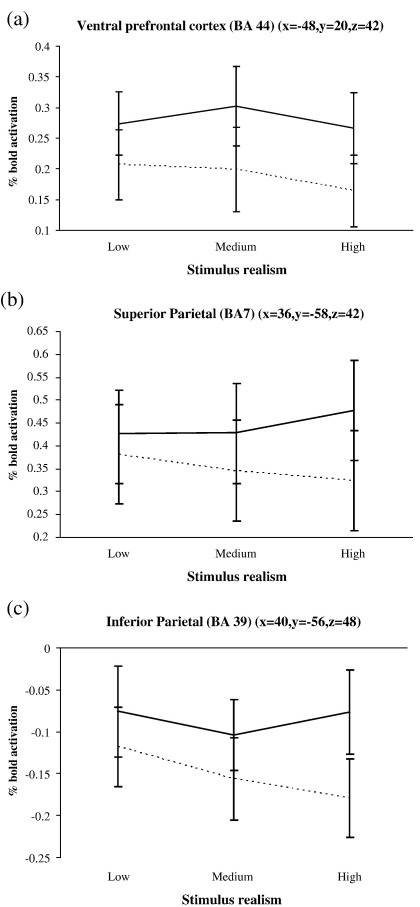
Bold activation % change (relative to baseline mean intensity) for (a) the ventral premotor cortex (BA 44); (b) the superior parietal cortex (BA 7); and (c) the inferior parietal cortex, over the different stimulus realism conditions. Dotted line indicates human instruction, solid line indicated computer instruction. Stimulus realism has been collapsed into low, medium and high. Standard error bars are shown.

A similar pattern of activation to BA 32 was observed in the pre-SMA, revealing higher activity when instructions and stimuli were inconsistent (Fig. 5a). The SMA proper demonstrated a reverse pattern with greater activity when instructions and stimuli were consistent (Fig. 5b). BA 44 displayed greater activation for computer compared with human instruction (Fig. 6a). Both parietal areas (BA 7 and BA 39) were more active for computer instructions but also for inconsistent instruction and stimuli conditions (Figs. 6b–c).

## Discussion

The results clearly show that pre-cueing participants that point-light stimuli were either human or computer generated movements influences both behavioural and neural responses to these stimuli. These conclusions can be summarised as three main findings: (1) human instruction had greater influence on behavioural responses for low realism trials, (2) computer and human instructions resulted in different patterns of brain activation and (3) two distinct areas of the paracingulate cortex responded to human instruction.

### Behavioural findings

As expected, the proportion of trials rated as “human” increased with increasing stimulus reality, showing that the objective quality of the stimuli influences perceptual judgments. It appears that the human instruction had a larger effect on responding at the low stimulus reality levels, with participants rating more low reality trials as human if accompanied by a human instruction. Pilot data (with a sample size of 15) had suggested that the categorising phenomenon would be consistent across all stimulus realism levels (i.e., the interaction was not significant): that is, there was a main effect of category (trials more likely to be rated as a person moving if categorised “human”) and a main effect of stimulus realism (more likely to be rated as a person moving at higher stimulus realism levels). It is not clear why the pattern of results differed slightly in the fMRI task: this might reflect the difficulty of decision making in the unusual fMRI environment.

#### Instruction affects brain responses to identical stimuli

Human and computer instruction resulted in differential activation of brain networks, even though the visual stimuli were identical across both instruction categories. The human instruction condition resulted in greater activation of paracingulate cortex, medial temporal cortex, as well as primary and extrastriate visual cortex, whereas the computer instruction was associated with greater activity in the ventral and dorsal prefrontal cortex, the inferior and superior parietal lobes and inferior and middle temporal gyri. Our results are in agreement with Martin and Weisberg (2003) who observed that interpreting identical abstract shapes as either social or mechanical events produced different patterns of brain activation. Interpreting shape movement as “social” also resulted in activation in the amygdala and ventral medial prefrontal cortex, and the authors suggested that these areas play a role in interpreting the social stimuli. Adding to this suggestion, the activation seen in the orbitofrontal cortex, amygdala, hippocampus, and parahippocampal regions during the human instruction trials in our work may be due to visual recognition processes, with participants comparing the point-light images with stored memories of human movement in order to interpret the human related stimuli. These areas play a role in memory processes (Petrides, 2007), and amygdala and orbitofrontal cortex activation has been reported in point-light tasks where participants were asked to memorise the sequences (Bonda et al., 1996). However, in the case of low stimulus realism, the point-light stimuli would not have closely matched memories of human movement, leading to activation in the prefrontal cortex, pre-SMA and dorsal paracingulate cortex which may form a network for the interpretation of more ambiguous or contradictory movement stimuli. Activation of the pre-SMA has been reported when participants are required to imagine walking (Malouin et al., 2003) and it is possible that in the case of conflict between human instruction and stimuli, participants may have internally simulated the observed movements in an attempt to understand their structure. Indeed, if as recently suggested that the pre-SMA is involved in processing errors between different sensory modalities (Yomogida et al., 2010), one would expect the pre-SMA to be more active during the conflict trials where there would be a mismatch between output from the internal simulation and the observed movement.

The brain areas more strongly activated for the computer instruction coincide with those reported during observation of objects and artificial motion. Observing point-light tool motion produces greater activity in the middle temporal gyrus and medial fusiform areas, compared to observing point-light human motion (Beauchamp et al., 2003); likewise, forming semantic judgments about inanimate objects leads to activation of the middle and inferior temporal gyri (Chao et al., 1999; see Puce and Perrett (2003) for a review). Beauchamp et al. (2003) suggested that these areas preferentially process artificial motion and, consistent with their findings, our temporal area activation was exclusively located within the left hemishpere. During a paradigm where participants rated whether computer animated characters were moving in a biological or artificial manner, Chaminade et al. (2007) observed that trials rated as artificial were associated with greater activity in the ventral premotor and posterior parietal cortices than trials rated as biological. Such frontal and posterior parietal areas may be particularly important for analysing spatial and kinematic visual sequences as Tavares et al. (2008) observed greater activity in these areas when participants paid attention to the spatial and kinematics properties rather than the behavioural interactions of two moving shapes. In addition, observation of non-biological motion and objects frequently activates prefrontal cortex areas, possibly due to prediction of forthcoming events in a sequence (Chaminade et al., 2001; Schubotz and von Cramon, 2003, 2004; Wolfensteller et al., 2007). As the superior parietal cortex, precuneus and prefrontal regions are associated with attentive tracking of moving objects (Culham et al., 1998) it is also possible that the activity in these areas may reflect greater cognitive demand for making judgments in a task when a stimulus is considered to be of artificial rather than biological origin. We propose that activation during the computer instruction trials supports the hypothesis that brain areas involved in prediction and attention to spatial and kinematic sequences are recruited during observation of artificial movement, or in this case movement believed to be of artificial origin.

### Paracingulate activity responds to human instruction

Our findings that paracingulate activity differentiates task processing in human instruction from computer instruction trials is in keeping with our earlier predictions and with previous work demonstrating that this area responds to human or socially related cues (Gallagher et al., 2002; McCabe et al., 2001; Ramnani and Miall, 2003; Schultz et al., 2005; Tavares et al., 2008). The paracingulate cortex has also been associated with tasks that require ToM (Castelli et al., 2000; Frith and Frith, 2006; Gallagher and Frith, 2003; Gallagher et al., 2000), judging the characteristics of others (Mitchell et al., 2005a,b; Mitchell et al., 2002) or understanding the goals and intentions of another human (Chaminade et al., 2002; Gallagher et al., 2002; McCabe et al., 2001; Tavares et al., 2008). Importantly, our task did not directly involve mentalising or goal attribution, yet paracingulate activity was observed in response to instructions that a stimulus represented human movement.

It has previously been suggested that different regions within the paracingulate cortex may have different functions: ventral paracingulate cortex may predominate in self-referential tasks or when judging others perceived as similar to oneself, whereas dorsal areas respond more when judging dissimilar others (Jenkins et al., 2008; Mitchell et al., 2005b, 2006). Völlm et al (2006) have suggested that ventral paracingulate is involved in emulating affective valence – feelings, desires, and motivation – while dorsal paracingulate cortex deals with more abstract mentalising about beliefs and knowledge. In our task, both dorsal and ventral areas were activated differentially according to the nature of the human or computer instructions. Ventral paracingulate cortex appears to be mainly responsive to the human instruction (Fig. 4a), whereas the more dorsal region of the paracingulate cortex is active when the stimuli and instructions are inconsistent (Fig. 4b). These findings support the previous suggestion (Jenkins et al., 2008; Mitchell et al., 2005b; Mitchell et al., 2006) that ventral paracingulate encodes stimuli deemed to be similar to ourselves (i.e. stimuli were interpreted as being of human origin); whereas dorsal paracingulate cortex may process information from ambiguous scenarios where prior knowledge and stimulus content are not easily reconciled (perhaps analogous to the dissimilar others hypothesis). It is tempting to speculate that activity in the dorsal region may correspond to the behavioural interaction seen for low stimulus reality.

Recent behavioural work highlights a prominent role for top-down modulation during priming tasks and that the default mode for processing stimuli may be at the level of goals and intentions (Chong et al., 2009; Liepelt et al., 2008; Longo et al., 2008). Overall, our current results suggest that the paracingulate cortex may be responsible for this top-down attribution of goals or intentions to observed movements. Returning to our previous work examining the influence of instruction on interference (Stanley et al., 2007), the human belief instruction may have led the participants to view the ambiguous dot movement as the product of intentional action, thereby producing interference effects and that the paracingulate cortex may have played a key role in the attribution of goals or intentions to observed movements.

#### The effect of task instruction on mirror neuron areas and STS

Our task did not appear to cause activation of the mirror neuron areas during the human instruction or high stimulus reality conditions. The lack of modulation of mirror neuron areas by human instruction highlights that even though a movement may be perceived as human, this does not guarantee activation in mirror neuron areas.

These findings compliment new ideas currently permeating the mirror neuron literature. Recent work shows that mirror neuron areas respond to non-biological stimuli, suggesting that, rather than being responsive to biological actions exclusively, such areas may function more generally in the prediction of actions and events (Cross et al., 2009; Engel et al., 2008a,b; Gazzola et al., 2007; Schubotz 2007; Wheatley et al., 2007). It is possible that our current finding of ventral prefrontal cortex (BA 44 and 45) activity in the computer instruction trials could add to the above findings, as mirror neurons may be present in both these regions (Kilner et al., 2009). However, as this activation was located anterior and superior to documented mirror neuron areas (Buccino et al., 2004; Grezes et al., 2003; Koski et al., 2003) and the location of our inferior parietal activity was more posterior than parietal regions usually demonstrating mirror activity (Engel et al., 2008a; Grezes et al., 2003; Perani et al., 2001) the possibility of mirror neuron activity during the computer instruction requires further study. All together, these results support the theory that areas related to social understanding (such as the medial prefrontal cortex), as opposed to mirror neuron areas, underlie the perception of human agency (Wheatley et al., 2007).

It is also possible that the use of point-light stimuli, combined with the passive nature of the current task, may be responsible for the lack of mirror neuron activation in our task. It could be argued that point-light animations may not be sufficiently rich visual stimuli to engage mirror neuron activity, as previous studies contrasting point-light human actions against scrambled movements have frequently failed to reveal ventral premotor activity (Bonda et al., 1996; Peelen et al., 2006; Pyles et al., 2007; Servos et al., 2002; however, for exceptions to this see Saygin et al. (2004) and Vaina et al. (2001)). This hypothesis is reinforced by the apparent lack of a correlation between mirror neuron area activity and the stimulus realism dimension in our data; furthermore, no such relationship was observed in the localiser task where the distinction between human and scrambled sequences was greater. However, findings of mirror neuron activity when viewing abstract stimuli would appear inconsistent with this interpretation (Cross et al., 2009; Engel et al., 2008a). In regards to instruction, as with previous point-light studies (Bonda et al., 1996; Peelen et al., 2006; Pyles et al., 2007; Servos et al., 2002) our task emphasized observation as opposed to reproduction, and mirror neuron responses are generally enhanced during tasks that involve imitating, imagining or predicting a movement as opposed to just observing (Engel et al., 2008b; Grezes et al., 1998; Iacoboni et al., 1999; Koski et al., 2003; Zentgraf et al., 2005). Previous tasks that have also cued participants to attend to the behavioural or social aspects of moving abstract stimuli, have failed to report mirror neuron activation (Blakemore et al., 2003; Schultz et al., 2005; Tavares et al., 2008). As with our study, it is therefore unclear whether lack of mirror neuron activity is due to the absence of movement reproduction or due to the abstract nature of the stimuli, although once again the findings of mirror neuron activity during observation of abstract stimuli would support the reproduction theory. Indeed, Wheatley et al. (2007) found mirror neuron area activation when they combined contextual cues to induce a sense of animacy over moving absract shapes, with a mental simulation and reproduction task. It will be informative in future work to manipulate agency instructions during a task that involves more active simulation processes.

As expected, our results revealed that as stimulus realism increased, participants more frequently rated the animations as resembling a moving person. This stimulus realism dimension was positively correlated with activity in the primary and extrastriate visual cortex, fusiform gyrus and middle temporal gyrus. Activation within these regions is consistent with previous studies that have examined neural responses to point-light stimuli (Blake and Shiffrar, 2007; Grossman et al., 2000; Vaina et al., 2001) and increased animacy (Schultz et al., 2005), and suggests that these areas respond specifically to the biological content of the stimuli rather than being influenced by top-down instruction effects. A relationship between STS activity and stimulus realism was also apparent in this study, albeit at a reduced level of significance. Given that STS activity is reduced with scrambled point-light motion displays compared to the untampered motion displays (Bonda et al., 1996; Grossman et al., 2000; Grossman and Blake, 2002; Saygin et al., 2004), and that even our highest stimulus reality level was partially scrambled, it is possible that the range of realism levels used in the study may not have been sufficient to fully differentiate STS activity across realism categories. As a consequence of this overall reduction in STS activity, our findings that STS activity was not significantly different across the 2 categories and that STS activity appeared to be greater for human instruction trials where stimulus reality was high should be interpreted with caution. Previous studies present mixed evidence regarding bottom-up and top-down influences on STS activity, with some demonstrating little top-down influence (Chong et al., 2008; Pelphrey et al., 2003a,b; Schultz et al., 2005) and others suggesting a stronger influence (Iacoboni et al., 2001; Pelphrey et al., 2003a,b). Tavares et al. (2008) observed STS activity when participants were cued to attend to the behavioural compared to spatial movements of two moving circles, where these movements contained no biological kinematics. In our experiment, the combination of explicit instructions and clearer biological motion led to increased STS activation, which together with the aforementioned studies suggests a complex interplay between instruction and movement type that deserves further research.

## Conclusions

Our results have demonstrated that different brain areas are involved when participants are informed that an identical visual stimulus is either of a human or computer origin: for the human instruction trials, brain regions involved in emotion and mentalising are preferentially activated, whereas for the computer instruction trials, areas involved in analysing sequences and spatial components of stimuli are activated. The paracingulate cortex appears to be a key component of this “human network” and our findings indicate that the simple suggestion of human movement activates this area, even in a task that requires neither social judgments nor mentalising processes. This finding is consistent with recent work showing top-down influences of human/computer categorising on activity in the paracingulate cortex (Gallagher et al., 2002; McCabe et al., 2001; Ramnani and Miall, 2003; Schultz et al., 2005; Tavares et al., 2008), and we posit that the paracingulate may form part of a default neural system for processing stimuli interpreted to be of human origin, or which possess human characteristics (e.g. cartoon human characters, words describing human psychological states; (Gallagher et al., 2000; Mitchell et al., 2002). We suggest that a person's disposition towards a stimulus depends on an interplay between bottom-up processing of the stimulus and top-down task instructions encoded or processed by the paracingulate cortex. Further work is required to understand how this top-down modulation affects mirror neuron areas and STS.

## Figures and Tables

**Table 1:** Mean percentage of trials (with 95% confidence intervals) rated as “human” by trial label (computer or human), and stimulus realism level (low, moderate, high).

Factor	Level of factor	Mean percentage rated as “yes, looks like a person moving”	95% confidence interval
Label	Computer	49.6	38.4 to 60.9
Human	57.7	46.5 to 68.1

Stimulus realism level	Low	19.5	12.4 to 29.4
Moderate	48.6	39.1 to 58.2
High	87.2	82.1 to 91

**Table 2:** Brain areas correlated with greater stimulus realism.

Motion content: positive correlation					Coordinates (mm)		
Area	Cluster volume (mm^3^)	Cluster *P*	*Z*	Laterality	*x*	*y*	*z*
Extrastriate visual cortex (BA 19)	84,881	< 0.0001	5.67	R	50	− 76	12
Middle temporal gyrus (BA 39)			5.47	R	54	− 72	16
Extrastriate visual cortex (BA 18)			5.3	R	40	− 84	10
Fusiform gyrus (BA 37)			5.11	R	38	− 46	− 22
Primary visual cortex (BA 17)	44,100	< 0.0001	7.02	R	2	− 90	6
Primary visual cortex (BA 17)			6.19	L	− 2	− 96	8
Extrastriate visual cortex (BA 19)	43,181	< 0.0001	4.86	L	− 48	− 80	− 8
Cerebellar lobule VI			4.71	L	− 32	− 42	− 26
Inferior temporal gyrus/fusiform gyrus (BA 37)			4.49	L	− 42	− 44	− 26

**Table 3:** Brain areas correlated with decreasing stimulus realism.

Motion content: negative correlation					Coordinates (mm)		
Area	Cluster volume (mm^3^)	Cluster *P*	*Z*	Laterality	*x*	*y*	*z*
Primary visual cortex (BA 17)	8063	0.05	6.33	R	14	− 98	10

**Table 4:** Brain areas more active during human compared to computer instruction trials.

Human–computer instruction					Coordinates (mm)		
Area	Cluster volume (mm^3^)	Cluster *P*	*Z*	Laterality	*x*	*y*	*z*
Extrastriate visual cortex (BA 18)	31,153	< 0.0001	6.65	R	20	− 96	− 14
Primary visual cortex (BA 17)			6.15	R	18	− 102	− 6
Primary visual cortex (BA 17)	28,819	< 0.0001	5.88	L	− 22	− 102	4
Extrastriate visual cortex (BA 18)			5.68	L	− 26	− 94	− 2
Insula cortex (BA 48)	18,281	< 0.0001	3.79	L	− 38	− 18	0
Parahippocampal (BA 36)			3.7	L	− 22	− 6	− 26
Hippocampus (BA 20)			3.56	L	− 28	− 8	− 22
Rolandic operculum (BA 48)			3.51	L	− 48	− 20	16
Amygdala (BA 34)			3.47	L	− 24	2	− 16
Paracingulate cortex (medial superior frontal gyrus (BA 10))	15,056	0.001	4.09	R	12	64	12
Orbital gyri (BA 11)			3.62	R	6	56	− 14
Paracingulate cortex (medial superior frontal gyrus (BA 32))			3.38	R	10	52	26

**Table 5:** Brain areas more active during computer compared to human instruction trials.

Computer–human instruction					Coordinates (mm)		
Area	Cluster volume (mm^3^)	Cluster *P*	*Z*	Laterality	*x*	*y*	*z*
Ventral prefrontal cortex (BA 45)	128,400	< 0.0001	6.39	L	− 46	28	28
Ventral prefrontal cortex (BA 44)			6.23	L	− 48	20	42
Dorsal lateral prefrontal cortex (BA 46)			5.88	L	− 44	52	− 4
Ventral prefrontal cortex (BA 47)			5.69	L	− 44	46	− 10
Inferior parietal lobe (BA 39)	60,881	< 0.0001	7.62	L	− 44	− 64	40
Superior parietal lobe (BA7)			6.29	L	− 36	− 66	50
Middle frontal gyrus (BA 11)	42,731	< 0.0001	5.49	R	32	58	2
Ventral prefrontal cortex (BA 45)			5.4	R	52	28	30
Ventral prefrontal cortex (BA 47)			4.32	R	44	54	− 12
Ventral prefrontal cortex (BA 44)			4.18	R	48	26	42
Precuneus (BA 7)	41,831	< 0.0001	7.37	R	2	− 68	48
Cuneus			4.88	–	0	− 72	34
Precuneus (BA 7)			4.2	L	-6	− 68	38
Inferior parietal lobe (BA 39)	30,394	< 0.0001	5.91	R	40	− 56	48
Superior parietal lobe (BA39/7)			5.46	R	36	− 58	42
Cerebellum crus 1	30,075	< 0.0001	5.75	R	8	− 82	− 30
Cerebellar lobule VIIIA			3.76	R	36	− 66	− 58
Inferior temporal gyrus (BA 20)	16,631	0.0005	5.02	L	− 68	− 42	− 14
Lingual gyrus (BA 37)			3.64	L	− 26	− 48	0
Middle temporal gyrus (BA 21)			3.42	L	− 44	− 40	− 2
Inferior temporal gyrus (BA 37)			3.39	L	− 60	− 54	− 16

**Table 6:** Brain areas more active when category and stimulus reality were inconsistent.

Category and stimulus interaction: inconsistent					Coordinates (mm)		
Area	Cluster volume (mm^3^)	Cluster *P*	*Z*	Laterality	*x*	*y*	*z*
Prefrontal cortex (BA 9)	10,744	0.01	4.84	R	18	50	38
Paracingulate cortex (medial superior frontal gyrus (border of BA 9/10/32))			3.43	R	6	50	30
Pre-supplementary motor area (BA 8)			3.31	R	4	24	56
Inferior parietal lobe (BA 40)	8213	0.04	3.51	R	46	− 58	56
Inferior parietal lobe (BA 39)			3.5	R	48	− 56	48
Superior parietal lobe (BA 7)			3.33	R	38	− 74	52
Superior parietal lobe (BA 7)			2.74	R	34	− 56	48

**Table 7:** Brain areas more active when category and stimulus reality were consistent.

Category and stimulus interaction: consistent					Coordinates (mm)		
Area	Cluster volume (mm^3^)	Cluster *P*	*Z*	Laterality	*x*	*y*	*z*
Anterior parietal area (BA 2)	105,413	< 0.0001	5.25	L	− 32	− 44	66
Anterior parietal area (BA 3)			5.24	L	− 34	− 28	50
Primary motor cortex (BA 4)			5.15	L	− 38	− 28	60
Supplementary motor area (BA 6)			4.65	L	2	− 14	54
Insula cortex (BA 48)	25,969	< 0.0001	4.57	R	44	− 12	4
Superior temporal lobe (BA 22)			3.61	R	66	− 38	18
Extrastriate visual cortex (BA 18)	11,250	0.008	3.97	R	22	− 96	16
Extrastriate visual cortex (BA 19)			3.31	R	20	− 82	32
Extrastriate visual cortex (BA 19)	9244	0.03	3.81	L	− 50	− 76	12
Middle temporal gyrus (BA 37)			3.31	L	− 48	− 60	6
Extrastriate visual cortex (BA 18)	8288	0.05	3.19	L	− 4	− 92	18
Superior parietal lobe (BA 7)			3.16	L	− 20	− 74	46

## Appendix A Supplementary data

Supplementary material I.

Supplementary material II.

Supplementary material III.

## Footnotes

3 The proportions chosen were based on a behavioural pilot study performed with 15 participants (see Supplementary data I).

4 The non-parametric Wilcoxon signed ranks test was also calculated for these paired comparisons, as this test is indifferent to the transformation of the data. The results of these hypothesis tests were all consistent with the results of the paired *t*-tests.
