# Supplementary material for: How instructions modify perception: An fMRI study investigating brain areas involved in attributing human agency
Source: Neuroimage. 2010 Aug 1;52(1):389–400. doi: 10.1016/j.neuroimage.2010.04.025 (PMC2887490; doi:10.1016/j.neuroimage.2010.04.025)
Supplement: Supplementary file 1 — Supplementary material I. [file mmc1.doc]

Supplementary Figure III. Activation map detailing areas of greater activity during biological motion compared to scrambled motion contrast for the (a) sagittal view (b) coronal view and (c) axial view. Coordinates in MNI space are indicated on each figure. Colour bars indicate z score significance level, from of 2.3 (black) to 7 (red).

(a)


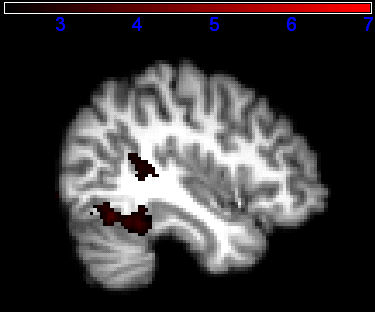


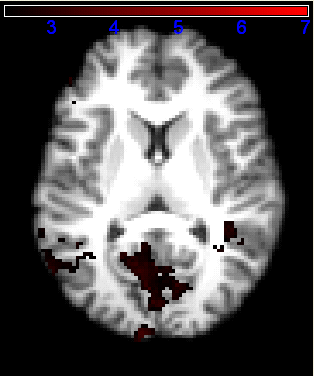

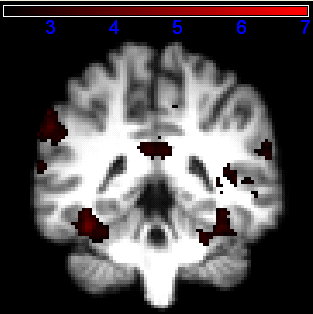
 (b) (c)

Superior temporal sulcus

y =-42

x =42

z =11
