# Supplementary material for: How instructions modify perception: An fMRI study investigating brain areas involved in attributing human agency
Source: Neuroimage. 2010 Aug 1;52(1):389–400. doi: 10.1016/j.neuroimage.2010.04.025 (PMC2887490; doi:10.1016/j.neuroimage.2010.04.025)

Supplementary data I: Behavioural pilot data.

15 participants (8 female, mean age 23.5 years, range from 19 to 32) completed a pilot version of the behavioural task. Trials were organised slightly differently from the final task– there were three larger blocks of trials, rather than 6 smaller blocks as in the fMRI study. All stimuli were identical to the main study. Participants in the pilot experiment were recruited in the same manner as participants in the main task, and were either paid for their time (1 hr) or received experiment participation credits in the School of Psychology.

Analysis of the behavioural data was conducted in the same manner as for the main study. Logit transformed data were used to compare the two category groups (human trial, computer trial) and the three different levels of stimulus realism (low, medium, high).

Results.

Analysis showed the human category trials were more likely to be responded to as “looks like a person moving” than were computer labelled trials, *F*(1,13) = 7.79, *p* = .015. Similarly, there was an increase in the likelihood of trials being labelled as a person moving with the increase in stimulus realism, *F*(2,26) = 181.38, *p* < .001. The interaction between label and realism was not significant, *F*(2,26) = 0.797, *p* = .43. The data for the pilot study are presented in Figure A1.

[Figure A1 on following page]

Figure A1. Proportions of trials rated as “looks more like a person moving than random”. Error bars show 95% confidence intervals (calculated on logit scale).


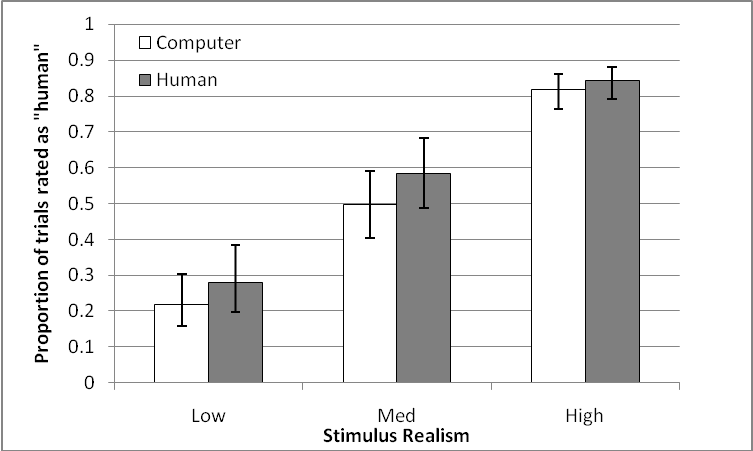

Supplement: Supplementary file 2 — Supplementary material II. [file mmc2.doc]
