# Supplementary material for: How instructions modify perception: An fMRI study investigating brain areas involved in attributing human agency
Source: Neuroimage. 2010 Aug 1;52(1):389–400. doi: 10.1016/j.neuroimage.2010.04.025 (PMC2887490; doi:10.1016/j.neuroimage.2010.04.025)
Supplement: Supplementary file 3 — Supplementary material III. [file mmc3.doc]

Supplementary material II

Task instructions

You are about to view a series of animations of moving dots. Some of these animations are recordings of actual human movement; other animations are computer generated random patterns of movement. At the start of each trial you will be informed of the type of animation you are about to view: after viewing the trial, you are required to judge whether your perception of that animation agreed with the type of trial it was. You will respond on this button box: the rightmost button is “strongly agree”, the next button is “slightly agree”, the next button is “slightly disagree” and the leftmost button is “strongly disagree”.

On trials preceded by the letter H (for human animation), you will be viewing an animation of actual recorded human movement. Each dot corresponds to a major joint or limb on an actor’s body. On these trials you are required to respond as to whether you perceived the moving dots as representing a person moving. Because this task is quite easy, we have added some noise to the dot positions to make the task more difficult. This means that on some trials the stimuli may not look like a person moving.

On trials preceded by the letter C (for computer generated animation), you will be seeing a randomly generated dot motion pattern. These patterns were created by the computer analyzing the speed and trajectory of human movement used in the first set of trials, and then generating a series of dot motion patterns based on this information. Therefore, sometimes these stimuli will appear similar to the “real” movements, and sometimes they will appear random. On these trials, you are required to respond as to whether you perceived the moving dots to be a random pattern of movement, rather than a moving person.
